# *Bacillus* sp. CSK2 produced thermostable alkaline keratinase using agro-wastes: keratinolytic enzyme characterization

**DOI:** 10.1186/s12896-020-00659-2

**Published:** 2020-12-14

**Authors:** Nonso E. Nnolim, Uchechukwu U. Nwodo

**Affiliations:** 1 SAMRC Microbial Water Quality Monitoring Centre, University of Fort Hare, Alice, 5700 South Africa; 2 Applied and Environmental Microbiology Research Group (AEMREG), Department of Biochemistry and Microbiology, University of Fort Hare, Private Bag X1314, Alice, 5700 South Africa

**Keywords:** Keratinase, *Bacillus* sp., Chicken feathers, Valorization, Thiol group, Laundry detergent

## Abstract

**Background:**

Chicken feathers are the most abundant agro-wastes emanating from the poultry processing farms and present major concerns to environmentalists. Bioutilization of intractable feather wastes for the production of critical proteolytic enzymes is highly attractive from both ecological and biotechnological perspectives. Consequently, physicochemical conditions influencing keratinase production by *Bacillus* sp. CSK2 on chicken feathers formulation was optimized, and the keratinase was characterized.

**Results:**

The highest enzyme activity of 1539.09 ± 68.14 U/mL was obtained after 48 h of incubation with optimized conditions consisting of chicken feathers (7.5 g/L), maltose (2.0 g/L), initial fermentation pH (5.0), incubation temperature (30 °C), and agitation speed (200 rpm). The keratinase showed optimal catalytic efficiency at pH 8.0 and a temperature range of 60 °C – 80 °C. The keratinase thermostability was remarkable with a half-life of above 120 min at 70 °C. Keratinase catalytic efficiency was halted by ethylenediaminetetraacetic acid and 1,10-phenanthroline. However, keratinase activity was enhanced by 2-mercaptoethanol, dimethyl sulfoxide, tween-80, but was strongly inhibited by Al^3+^ and Fe^3+^. Upon treatment with laundry detergents, the following keratinase residual activities were achieved: 85.19 ± 1.33% (Sunlight), 90.33 ± 5.95% (Surf), 80.16 ± 2.99% (Omo), 99.49 ± 3.11% (Ariel), and 87.19 ± 0.26% (Maq).

**Conclusion:**

The remarkable stability of the keratinase with an admixture of organic solvents or laundry detergents portends the industrial and biotechnological significance of the biocatalyst.

**Supplementary Information:**

The online version contains supplementary material available at 10.1186/s12896-020-00659-2.

## Background

Keratinases (EC 3.4.21/24/99.11) are inducible proteolytic enzymes that mediate the bioconversion of insoluble keratinous protein into smaller organic molecules including functional peptides and amino acids [1]. The enzymes have been reported to disintegrate keratin either in synergy [2], or isolation [3] of sulfitolytic systems. Keratinases are mostly serine or metallo-class of protease, and are distinct from classic proteases owing to their ability to degrade keratin which is the major structural constituent of avian feathers, hair, hooves, nails and horns.

Keratinous biomass are generated in significant quantities from commercial slaughterhouses, and poultry and leather processing industries [4]. The recalcitrant tendency of keratinous residues poses disposal challenge which leads to a localized accumulation, and that ultimately constitute environmental nuisance [1]. Globally, avian feathers are generated in large quantities annually, and the trend should be in the upward trajectory as the demand for white meat rises with the ever-growing world population [5].

Chicken feathers account for about 5–7% of the total weight of a chicken [6]. Therefore, the keratinous waste emanating from poultry processing farms are enormous and presents major concerns to the environmentalists. In the Republic of South Africa, about two hundred and thirty million kilograms (230 × 10^6^ kg) of chicken feathers are generated per annum [7], due to the promotion of local productions and imposition of anti-dumping duties on importation of poultry meats from Brazil and other top players in the industry [8]. Since the demand for these agro residues is unfortunately low, feathers are predominantly landfilled or incinerated [9]. A number of approaches have been used to add economic value to the protein-rich poultry feathers, and that includes the adoption of endergonic–mechanical method for the conversion of feathers into feedstuffs (feather meal) by some agro-industry [10]. The valorization process is sometimes used for the extraction of pure keratin from feathers via the application of organic/inorganic chemicals [11]. However, all this approach is expensive and not environmentally friendly. On a similar note, the physical-mechanical valorization approach always, is, saddled with loss of essential constituents in the feather meal [12].

The bio-based approach for the valorization of poultry feathers (keratin-rich polymer) represents an efficient route for novel products. Therefore, it portends an effective and sustainable reintegration of the agro residues into a value chain for the bio-economy [13]. Consequently, some microbes including bacteria, actinobacteria and fungi have expressed keratinolytic potentials [14, 15]. The bacteria have been the most explored, and *Bacillus* spp. are the lead keratinase producers [16]. The biodegradation of keratinous wastes may serve a multiple beneficial purpose including the production of economically viable microbial metabolites, the generation of degradation products with economic value, and the promotion of a cleaner environment [17]. Extracellular keratinase production by microorganisms differs significantly because of the salient variations of keratinolytic microbial strains. The optimization process, either through classical or statistical method, has been employed to enhance keratinase yield from various wild bacterial isolates, as keratinase production is grossly influence by physical and nutritional factors. The construction of optimal physico-chemical conditions improved the keratinase production from *Alcaligenes* sp. AQ05–001 by a 10-fold increase [17]. Similarly, Tiwary and Gupta [18] reported an enhanced extracellular keratinase secretion from a feather-degrading *Bacillus licheniformis* ER-15 after the optimization of the process conditions.

Keratinases have potential applications in various sectors including the leather production, detergent formulation, pharmaceuticals and biomedicine [12, 14], not only because of their catalytic efficiency, but also due to sustainable production on a cost-effective renewable resource. The ability of keratinases to hydrolysis both the soluble and insoluble proteinaceous stains; and robustness in the presence of chemical agents promoted their industrial significance as bio-additive for detergent formulation [19]. Moreover, keratinases possess boisterous tendencies suitable for green technology. Therefore, continuum in the search for a novel keratinase producer from diverse environment is an imperative. This study was undertaken to enhance the extracellular keratinase activity of *Bacillus* sp. CSK2 via the process conditions optimization. The properties of the extracellularly secreted keratinase were determined, and the putative keratinase coding gene was amplified, sequenced and the sequence analyzed for novel properties.

## Results

### Optimization of physico-chemical process

**Fig. 1:**
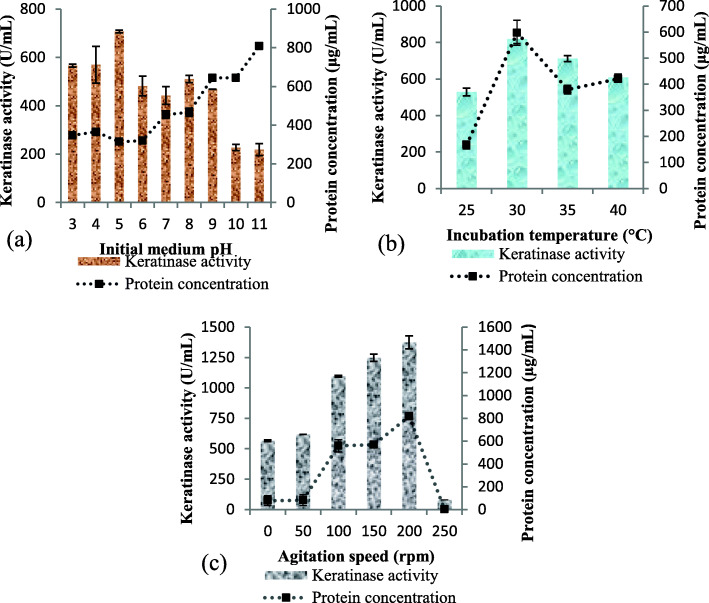
Effect of **a** initial medium pH (3.0–11.0) **b** incubation temperature (25–40 °C) and **c** agitation speed (0–250 rpm) on extracellular keratinase production by *Bacillus* sp. CSK2

The initial medium pH was varied from 3.0 to 11.0 and results showed that *Bacillus* sp. CSK2 significantly produced extracellular keratinase at a broad range of pH (3.0–9.0), with the maximum activity of 707.27 ± 5.14 U/mL at pH 5.0 (Fig. 1a). Above pH 9.0, the enzyme production declined considerably, with keratinase activity of 227.27 ± 12.85 U/mL and 219.09 ± 24.42 U/mL at pH 10.0 and 11.0, respectively. The protein concentration was quantified from the cell-free broth, and the results indicated that the protein content was relatively constant at weakly acidic conditions (pH 3.0–6.0). The concentration of protein signifcantly increased after pH 6.0, with the maximum concentration of 808.03 ± 14.48 μg/mL at pH 11.0 (Fig. 1a).

The incubation temperature of the fermentation flasks was varied and the results indicated that *Bacillus* sp. CSK2 produced keratinase in all the evaluated temperatures (25 – 40 °C). The optimum keratinase activity of 819.09 ± 24.42 U/mL was obtained at 30 °C (Fig. 1b). Above 30 °C, the enzyme production decreased consistently from 35 °C to 40 °C, with respective keratinase activity of 711.82 ± 16.71 U/mL and 609.09 ± 17.99 U/mL. The protein concentration showed similar pattern as enzyme activity, with the maximum concentration of 596.87 ± 47.91 μg/mL at 30 °C.

The effect of static condition and different agitation speeds on keratinase production by *Bacillus* sp. CSK2 was assessed. The results showed that the isolate produced enzyme at static condition (565.45 ± 7.71 U/mL), but the activity was significantly enhanced by the agitation speed, with the highest enzyme activity of 1374.54 ± 53.99 U/mL obtained at 200 rpm (Fig. 1c). Similarly, the maximum protein concentration of 819.06 ± 32.31 μg/mL was obtained at 200 rpm. Beyond optimum, both enzyme activity and concentration of protein showed drastic decrease at 250 rpm.

**Fig. 2:**
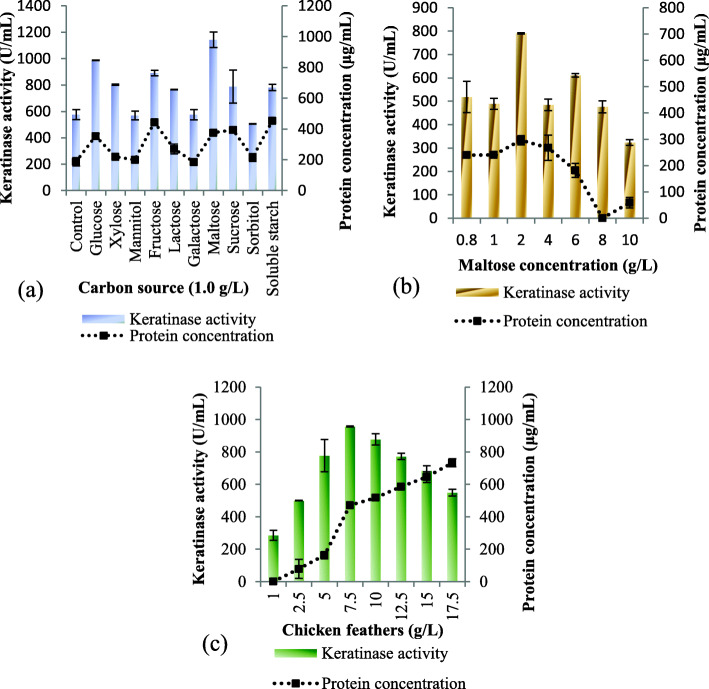
Effect of **a** carbon source supplementation (1.0 g/L) **b** maltose concentration (0.8–10.0 g/L) and **c** chicken feather concentration (1.0–17.5 g/L) on extracellular keratinase production by *Bacillus* sp. CSK2

Subsequently, the fermentation medium was supplementation with various carbon sources, and the extracellular keratinase activity of *Bacillus* sp. CSK2 was promoted in the presence glucose, xylose, fructose, lactose, maltose, sucrose and soluble starch when compared to the control with respective enzyme activity of 987.27 ± 2.57 U/mL, 801 ± 5.14 U/mL, 890.90 ± 20.57 U/mL, 765.45 ± 2.50 U/mL, 1142 ± 57.85 U/mL, 788.18 ± 124.70 U/mL and 780.90 ± 24.43 U/mL (Fig. 2a). The maximum stimulatory effect was obtained after maltose supplementation. The supplementation of the medium with mannitol, galactose and sorbitol did not produce any conspicuous effect when compared to the control. The protein concentration fluctuated across the different carbon sources employed with the highest concentrations of 443.23 ± 0 μg/mL and 452.68 ± 2.22 μg/mL recorded against fructose and soluble starch, respectively. The influence of maltose concentration was further assessed, and the finding indicated that 2 g/L was optimal for keratinase activity of *Bacillus* sp. CSK2 (Fig. 2b). Also, the concentration of the protein was the maximum at 2.0 g/L, and subsequently decreased with higher maltose concentration.

The influence of various concentrations of chicken feathers on extracellular keratinase production by *Bacillus* sp. CSK2 was further studied. The obtained results highlighted that increasing concentration of chicken feathers from 1.0, 2.5, 5.0 to 7.5 (g/L) resulted in stepwise increase in extracellular keratinase activity from 285.45 ± 30.85 U/mL, 499.09 ± 1.28 U/mL, 777.27 ± 98.99 U/mL, to 957.27 ± 3.85 U/mL, respectively (Fig. 2c). Beyond the optimum concentration (7.5 g/L), extracellular keratinase production dropped in a consistent manner with increasing chicken feathers concentration; presenting the least enzyme activity of 549.09 ± 20.57 U/mL at 17.5 g/L. The medium protein content increased as higher amounts of chicken feathers were utilized for the fermentation and the maximum concentration of 733.17 ± 24.51 μg/mL was recorded at 17.5 g/L.

### The time course study of keratinase activity

**Fig. 3:**
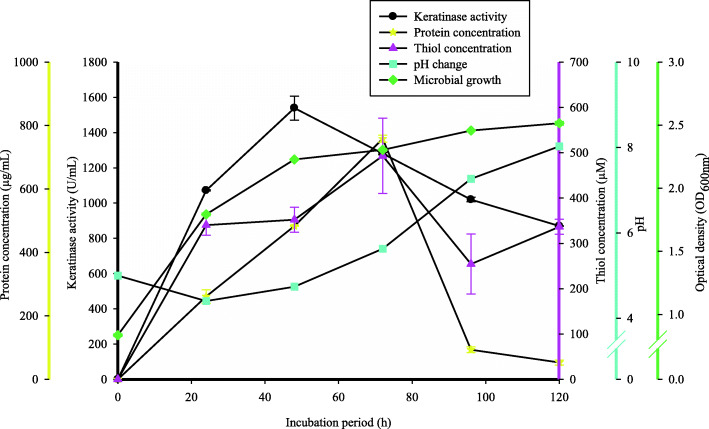
The time course study of keratinolytic activity of *Bacillus* sp. CSK2 at optimized conditions over the incubation period of 120 h. Aliquots were periodically (24 h) withdrawn in an aseptic condition to determine keratinase activity (black), protein concentration (yellow), thiol concentration (pink), pH change (cyan) and cell growth (green)

The time course of keratinase production by *Bacillus* sp. CSK2 was evaluated for 120 h. Keratinase production began during the exponential growth phase of the isolate and subsequently peaked at 48 h of incubation period with keratinase activity of 1539.09 ± 68.14 U/mL (Fig. 3). Beyond 48 h, the enzyme activity consistently declined, reaching 869.99 ± 1.28 U/mL at 120 h. The protein and thiol concentrations peaked at 72 h with respective concentrations of 758.39 ± 11.14 μg/mL and 492.98 ± 82.99 μM (Fig. 3). Extension of the fermentation period resulted in drastic decrease of the both protein and thiol concentrations. The evaluation of pH change over the course of fermentation showed that the fermentation medium pH dropped after 24 h of incubation from initial value of 5.0 to 4.4 ± 0.12 (Fig. 3); thereafter, it increased consistently following the extension of cultivation period with the maximum value of 8.03 ± 0.02 at 120 h.

### Enzyme characterization

#### Effect of pH on keratinase activity and stability

**Fig. 4:**
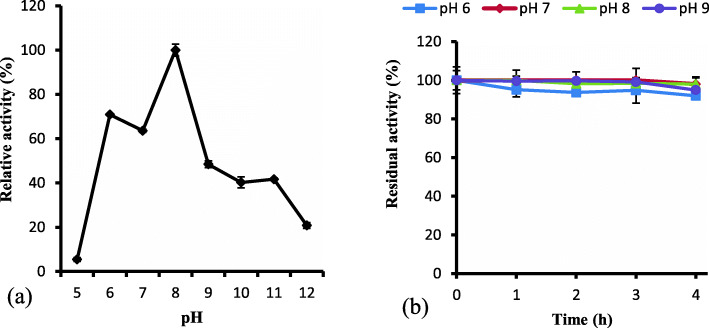
**a** Effect of pH (5.0–12.0) on keratinase activity. The highest enzyme activity was obtained at optimum pH 8.0. **b** Determination of pH stability of keratinase after 4 h preincubation at pH 6.0, 7.0, 8.0 and 9.0

The evaluation of the pH influence on keratinase activity indicated that the keratinolytic enzyme was catalytically active from weakly acidic condition to alkaline condition (Fig. 4a). The keratinase displayed the optimum activity at pH 8.0. It barely showed activity at pH 5.0; while it presented about 48% relative activity at pH 9.0 and further declined with increasing alkalinity. Subsequently, the evaluation of the keratinase stability at different pH values (6.0–9.0) showed that the enzyme was remarkably stable, retaining 91.97 ± 0.15, 97.97 ± 3.36, 97.99 ± 3.83 and 94.96 ± 1.29 (%) of the original activity at respective pH 6.0, 7.0, 8.0, and 9.0 after 4 h of preincubation (Fig. 4b).

### Effect of temperature on keratinase activity and stability

**Fig. 5:**
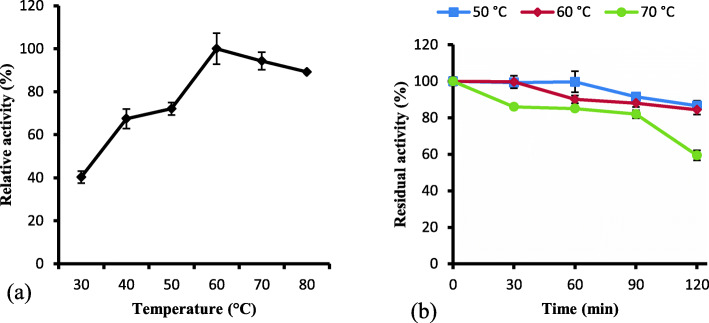
**a** Effect of temperature (30 – 80 °C) on keratinase activity. The optimal temperature for keratinase activity was determined to be in the range of 60 – 80 °C. **b** Determination of the thermostability of keratinase after 2 h of preheating at 50 °C, 60 °C and 70 °C

The temperature profile of *Bacillus* sp. CSK2 keratinase was presented in Fig. 5. The enzyme was active at a broad temperature range (30–80 °C), and the activity increased following the rise in temperature with the highest activity at 60 °C (Fig. 5a). However, there was no significant difference between keratinase activity at 60 °C and 70 °C or 80 °C as the relative activities at latter temperatures were 94.30 ± 4.10% and 89.22 ± 0.15%, respectively.

Furthermore, the keratinase solution was subjected to heat pretreatment at 50 °C, 60 °C and 70 °C for 2 h, and the findings indicated that the study enzyme was remarkably stable with respectively residual activity of 86.65 ± 2.76%, 84.37 ± 2.53%, and 59.45 ± 2.76% (Fig. 5b).

### Effect of various chemical agents on stability of keratinase

The keratinase showed complete inhibition with an admixture of metallic ions chelators, EDTA and 1,10-phenanthroline, but retained 89 ± 6.22% of the original activity when pretreated with PMSF for 60 min (Table 1). Among all the reducing agents, 2-mercaptoethanol had stimulatory effect on the residual activity (111 ± 0.54%) of the keratinase; while sodium thioglycolate, dithiothreitol and sodium sulfite affected the enzyme stability with residual activities of 64 ± 2.70%, 69 ± 0.09% and 78 ± 3.61%, respectively (Table 1). Furthermore, the enzyme was remarkably stable (103 ± 3.52%) at 1% (v/v) hydrogen peroxide (Table 1). The organic solvent impacted variably on the stability of the keratinase with stimulatory effect demonstrated after DMSO pretreatment (114 ± 5.86%). Contrarily, acetonitrile and propan-2-ol prompted lower stability of the enzyme. The study keratinase showed remarkable stability after pretreatment with non-ionic surfactants, triton X-100 (99 ± 2.16%) and tween-80 (182 ± 1.98%). While the anionic surfactant, SDS caused a reduction of enzyme activity at 5 mM (Table 1).

**Table 1:** Effect of various chemical agents on keratinase stability

Chemical agent	Concentration	Residual activity (%)
None	**–**	100 ± 4.24^d^
EDTA	5 mM	0 ± 0^a^
1,10-phenanthroline	5 mM	0 ± 0^a^
PMSF	5 mM	89 ± 6.22^c^
Sodium thioglycolate	5 mM	64 ± 2.70^b^
Dithiothreitol	5 mM	69 ± 0.09^b^
Sodium sulfite	5 mM	78 ± 3.61^c^
2-Mercaptoethanol	5 mM	111 ± 0.54^e^
Hydrogen peroxide	1% (v/v)	103 ± 3.52^d^
Dimethyl sulfoxide	1% (v/v)	114 ± 5.86^e^
Acetonitrile	1% (v/v)	61 ± 3.34^b^
Propan-2-ol	1% (v/v)	79 ± 3.97^c^
Sodium dodecyl sulfate	5 mM	77 ± 4.51^c^
Triton X-100	1% (v/v)	99 ± 2.16^d^
Tween-80	1% (v/v)	182 ± 1.98^f^

Superscript letter(s) a, b, c, d, e and f down the column were used to indicate significant difference at *P* < 0.05

### Effect of various metallic ions on the stability of keratinase

The effect of different monovalent, divalent and trivalent metal ions on the enzyme stability was investigated, and the results were presented in Table 2. All the metal ions employed negatively impacted on the stability of the enzyme, with more drastic effect obtained in the presence of trivalent metal ions, Al^3+^ (13 ± 1.35%) and Fe^3+^ (12 ± 0.36%).

**Table 2:** Metal ion effect on the stability of keratinase

Metal ion	Concentration (mM)	Residual activity (%)
None	–	100 ± 4.24^f^
Li^+^	5	71 ± 1.71^e^
Na^+^	5	71 ± 1.53^e^
K^+^	5	71 ± 4.78^e^
Ag^+^	5	69 ± 3.69^e^
Ca^2+^	5	69 ± 2.34^e^
Mg^2+^	5	68 ± 4.42^e^
Mn^2+^	5	74 ± 1.35^e^
Zn^2+^	5	69 ± 0.81^e^
Cu^2+^	5	45 ± 2.52^bc^
Fe^2+^	5	73 ± 1.62^e^
Hg^2+^	5	43 ± 2.07^b^
Ba^2+^	5	55 ± 3.61^d^
Co^2+^	5	50 ± 1.98^cd^
Al^3+^	5	13 ± 1.35^a^
Fe^3+^	5	12 ± 0.36^a^

Superscript letter(s) a, b, c, d, e and f down the column were used to indicate significant difference at *P* < 0.05

### Laundry detergent effect on the stability of keratinase

**Fig. 6:**
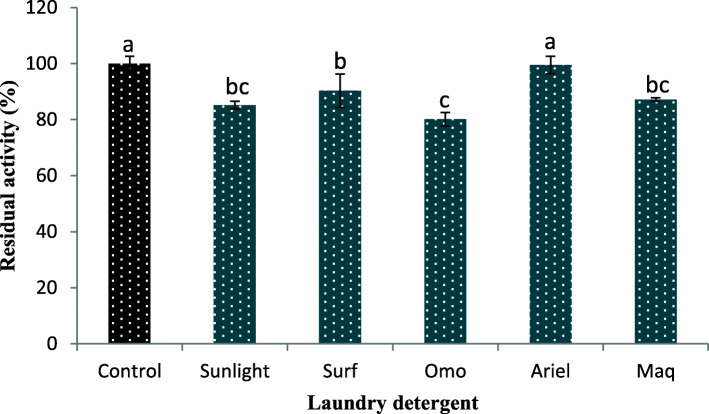
Laundry detergent impact on keratinolytic protease stability. Letters a, b and c indicate significance difference among the various treatments and points without similar letter(s) are different statistically (*P* < 0.05)

The results from the detergent stability study showed that the crude keratinase maintained remarkable stability in the various laundry detergents employed, with residual activity of 85.19 ± 1.33, 90.33 ± 5.95, 80.16 ± 2.39, 99.49 ± 3.11 and 87.19 ± 0.26 (%) for Sunlight, Surf, Omo, Ariel and Maq, respectively (Fig. 6). There was no significant difference (*P* > 0.05) between the residual keratinase activity after the Ariel pretreatment and control (enzyme solution preincubated with tap water only).

### Molecular characterization of keratinase

The gel picture of keratinase encoding gene amplified from *Bacillus* sp. CSK2 showed band size of 1104 bp (supplementary file). The nucleotide sequence analysis indicated that the putative keratinase gene has 92 and 93% identity with keratinase genes of *Bacillus thuringiensis* S3KUBOT (KX155576) and *Bacillus cereus* Wu6 (HQ694987), respectively. The nucleotide sequence with a G + C content of 39.4% was submitted to the GenBank with the accession number MT268136.

**Fig. 7:**
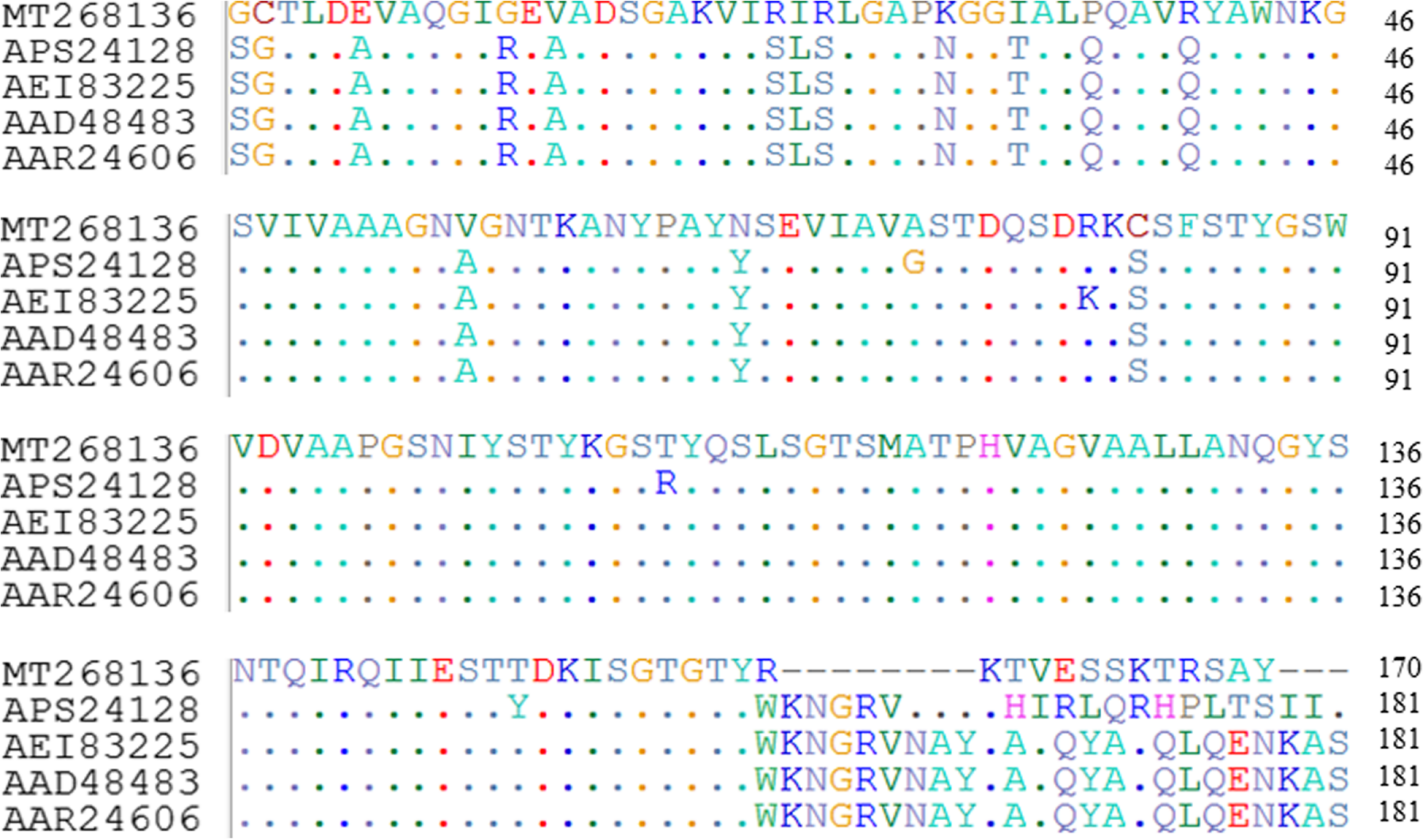
Multiple amino acid sequences alignment of *Bacillus* sp. CSK2 keratinase with *B. thuringiensis* S3KUBOT keratinase (APS24128), *B. cereus* Wu6 keratinase (AE183225), *B. thuringiensis* alkaline protease A (AAD48483), and *B. thuringiensis* alkaline protease A (AAR24606). The dots indicate similar residues between the study keratinase sequence and other sequences from the protein database

Figure 7 shows the alignment of amino acid sequence deduced from the partial nucleotide sequence with other related keratinase and alkaline protease sequences of *B. cereus* group (sensu *lato*), including *B. thuringensis* S3KUBOT keratinase (APS24128), *B. cereus* Wu6 keratinase (AE183225), *B. thuringiensis* alkaline proteases (AAD48483) and (AAR24606). The keratinase under investigation showed high sequence homology with other related sequences obtained from the protein database. The dots indicated similar amino acid residues down the column between the study sequence and the reference sequences; while the points of variation are shown by the retention of amino acid residues on the reference sequences (Fig. 7).

## Discussion

The pH optimum for the extracellular keratinase activity of *Bacillus* sp. CSK2 was previously demonstrated by a few *Bacillus* spp. [20, 21]. The optimal extracelular keratinase production at pH ≤ 6.0 has been sparcely documented, as acidic condition has been described as the limiting factor for the expression of keratinase by most of *Bacillus* spp. [22, 23]. Generally, keratinolytic bacteria have been described to optimally secrete keratinase at pH ranging from weakly acidic condition to alkaline spectrum [24]. The higher protein concentration observed at alkaline condition may be attributed to either the elevated production of other proteins that are not keratinase by bacterial isolate or libration of soluble proteins from the feather keratin directed by the basic condition of the medium [25]. It is worthy of note that pH is an important factor that contributes significantly to the regulation of microbial growth, plasma membrane integrity and transport proteins functionality [24]. Consequently, it influences microbial metabolism and metabolites production via some cascade mechanisms. The ability of *Bacillus* sp. CSK2 to considerably produce extracellular keratinase at an extended pH spectrum underpins its biotechnological and indrustrial relevance.

The findings from the temperature optimization suggest that *Bacillus* sp. CSK2 is a mesophilic bacterium, and this attribute of strain CSK2 is energy saving and economically attractive [26]. This is consistent with other reports on keratinolytic bacteria that presented optimal keratinase production at mesophilic conditions [22, 26, 27]. However, some bacterial strains have been reported to optimally express keratinase at relatively higher temperature [28], including the extremophile – *Fervidobacterium islandicum* AW-1 [29].

The agitation speed optimal is peculiar to bacterial isolate; as such variable optimal agitation rates have been reported for the maximum extracellular keratinase productions [30, 31]. Agitation speed promotes microbial metabolism by ensuring optimal oxygen transfer and effective microbial cell-substrate interactions through medium homogeneity which subsequently enhances cell growth and enzyme production [32]. Nonetheless, higher agitation speed prompts shear forces which affect cell membrane integrity and productivity [32]. Keratinase production by other strains of *Bacillus* spp. was also enhanced at similar agitation condition [27, 33].

Basically, microbial keratinase production is an inducible process, and it begins when the microbial cells significantly depend on keratinous biomass for homeostasis [21]. The improved enzyme production obtained when the fermentation media were supplemented with low concentration of various assimilable carbon sources may be attributed to the facilitation of the bacteria growth encouraged by the utilizable carbon sources. Simultaneously, the bacterial cells solely relied on the chicken feathers for nitrogen which invariably prompted the upregulation of the keratinase expression, yielding to the significant extracellular enzyme concentration for efficient keratin hydrolysis [34]. The peculiarity of microorganisms influences their preference to carbon sources, hence the observed variation in keratinase production with various supplemented carbon sources [17]. The positive influence of the various carbon sources supplemented has been reported in similar studies [18, 26, 35].

As reported in similar studies, the maximum keratinase production by some bacterial species was obtained using the concentration of chicken feathers that ranged from 5.0 g/L to 10.0 g/L [17, 23, 26, 27]. Conversely, keratinase production by *Stenotrophomonas maltophilia* [35] and *Xanthomonas* sp. P5 [36] was optimum in media formulated with 2.0 g/L and 1.0 g/L of chicken feathers, respectively. As an inducible enzyme, it was expected that the extracellular keratinase activity would have increased with higher concentrations of chicken feathers, but that did not happen, instead protein concentration increased consistently. This observed trend of high protein content at higher concentration of chicken feathers may be attributed to the medium accumulation of soluble proteins emanating from keratin hydrolysis by active extracellular keratinase. These assimilable products would prompt the down-regulation of keratinase expression via product inhibition mechanism [15], hence the decrease in the concentration of extracellular keratinase. The finding is consonant with the previous reports [26, 27].

The findings from the time course study suggest that keratinase production by *Bacillus* sp. CSK2 was associated with primary metabolism [27]; and the keratinase production in optimized conditions was enhanced 3.4-folds when compared to the unoptimized condition [16]. Similarly, He et al. [37] reported the highest keratinase production of 21.6 U/mL by *B. subtilis* 8 at 48 h of fermentation. Likewise, *S. maltophilia* R13 presented optimum extracellular keratinase activity of 82.3 ± 1.0 U/mL after 48 h of incubation period [35]. The efficient keratinase secretion potential of *Bacillus* sp. CSK2 within the established short time frame portends its relevance in industry and biotechnology. Keratin is a recalcitrant biopolymer stabilized by cystine disulfide cross linkages. The detection of thiol groups in the fermentation broth may indicate the reduction of densely populated disulfide bonds of feather keratin into bioavailable products, and the thiol concentration may suggest the biocatalytic efficiency of the keratinolytic and/or sulfitolytic systems [24, 38]. The thiol concentration determined in the present study is significantly higher than that reported in the previous studies, where maximum thiols of 44.5 ± 1.8 μM [35], 82 μM [39], and 5.4 ± 0.4 μM [37] were obtained at 24 h, 72 h, 96 h and 120 h of incubation period, repectively. This attribute of *Bacillus* sp. CSK2 highlights its dexterity in the valorization of keratinous wastes. Bioconversion of keratin-rich biomass potentiates the ammonification of a culture medium through deamination of free amino groups containing degradation products and this process tends to shift the medium pH toward alkalinity [18, 38]. The initial slight decrease of culture pH may be attributed to the organic acids emanating from the primary metabolic activity of the isolate. The growth of keratinolytic *B. lichenifoirmis* ER-15 [18] and *Xanthomonas* sp. P5 [37] on chicken feathers lowered the medium pH in like manner.

Change in pH instigates protonation or deprotonation of protein side chain residues which may affect enzyme – substrate binding and product formation through alteration of the enzyme conformation [39]. As observed from the study keratinase, more drastic effect of pH on biocatalyst may lead to complete loss of activity. The pH optima of bacterial keratinases have been extensively documented within the range of 7.0 to 9.0 [24, 40]. However, some reports on alkalophilic keratinase abound with pH optima ≥11.0 [4]. The pH stability of the keratinase may be likened to that demonstrated by the alkaline keratinase from *B. thuringiensis* MT1 [12] and *Bacillus subtilis* RSE163 [41]. Conversely, *Bacillus* sp. CSK2 keratinase pH stability was superior to that reported for *Bacillus altitudinis* RBDV1 keratinase [42]. Therefore, the considerable pH stability of this enzyme supports its potential application in detergent formulation where most promising proteases operate between pH range of 7.0 and 10.0 [39].

The broad thermal activity exhibited by the study enzyme is consistent with most of the documented optimal temperatures of keratinases [24]. The maximum biocatalytic efficiency of *B. licheniformis* RPK keratinase was obtained at 60 °C [43]. However, extremophilic keratinase from *F. islandicum* AW-1 was optimally active at 100 °C [29]. The broad temperature tolerance of the keratinase from *Bacillus* sp. CSK2 suggests the amplitude of its relevance in biotechnology and industry. An alkaline keratinase from *Bacillus halodurans* PPKS-2 displayed similar thermostability pattern as reported in the present study [44]. Contrariwise, *Bacillus pumilus* A1 keratinase retained 30.3% of the original activity after 1 h of heating at 60 °C [45]. Additionally, thermostability profiles of keratinases from *Bacillus* spp. showed that at 60 °C and 70 °C after 1 h of preincubation, the residual activities of 30 and 7% were retained by BPker, and 37 and 15% were retained by BAker [46]. The temperature stability of the presently characterized keratinase supports its future applicability in green technological processes.

The sensitive of the enzyme under investigation to the protease inhibitors suggests that it belongs to the metallo-class of keratinase [47–49]. The removal of inherent metallic ions from proteolytic enzyme by chelating agents perturbs the activity of the protein by destabilizing the catalytic conformation which subsequently affects enzyme-substrate binding and product formation [45, 50]. Metallo-keratinase from *Chryseobacterium* sp. was catalytically stimulated by 2-mercaptoethanol [50]. Conversely, stability of metallo-keratinase from *Microbacterium* sp. kr10 was greatly affected by 0.5% (v/v) 2-mercaptoethanol, retaining about 14% of the original activity [51]. Generally, reducing agents have been implicated in the perturbation of keratinase stability by reducing the intra-molecular disulfide bonds that are involved in structural stabilization [2, 52]. The considerable stability of the keratinase in oxidizing agent portends its industrial potentials including the production of bleach based detergents and similar stability pattern was reported for keratinase from *B. subtilis* RSE163 [41]. The biocatalysis promotion by DMSO and destabilization of keratinase catalytic efficiency by various organic solvents were reported previously [42, 46, 50, 53]. It is worthy of note that tween-80 promoted keratinase activity by 82%, and this property of the study enzyme is similar to that reported for *Brevibacillus* sp. AS-S10-II keratinase which has been advanced as good candidate for detergent formulation [52].

Metallo-keratinases have shown to display variable metallic ions tolerance [47, 48, 51]. Zhang et al. [49] reported that keratinase from *Brevibacillus parabrevis* was selectively activated at 1 mM metal ions, and further increase in the concentration of metal ions up to 5 mM strongly impacted the stability of the enzyme. Additionally, *Bacillus subtilis* KD-N2 keratinase was significantly inhibited by all the metal ions tested at 5 mM final concentration [53]. Metallic ions influence the activity of a protein by allosteric regulation through complexation with essential residues which induces disorientation of the proper enzyme conformation that encourages substrate binding [6]. Therefore, at high concentrations of metal ions, the catalytic efficiency of metallo-keratinase may be hindered via the process of ionic tethering and electrostatic interaction [50, 54].

Keratinolytic protease from *B. halotelerans* CT2 demonstrated similar significant residual activity after pretreatment with selected commercial laundry detergents [55]. Likewise, crude keratinase from *B. pumilus* GRK showed considerable residual activity in the range of 80.32 ± 0.05–93.27 ± 0.16% after 60 min of pretreatment with 0.7% (w/v) of the various laundry detergents used [27]. Conversely, the stability of the study enzyme was remarkably higher than *Paenibacillus woosongensis* crude keratinase that showed a range of 48.1–70.4% residual activity under similar treatment conditions [19]. Recently characterized alkaline protease from *Bacillus safensis* retained 75% of the original activity after preincubation with Ariel (7.0 mg/mL) [56]. The detergent ingredients including fiber brighteners, foam regulators, bleaching agents, re-deposition agents, surfactants, softening builders, among others have been reported to affect the stability of detergent’s endogenous protease [27, 52]. Therefore, the ability of the study keratinase to maintain significant stability in the presence of the various laundry detergents tested suggests its potential candidacy in detergent formulation.

The successful amplification of the keratinase gene therefore buttressed the keratinolytic efficiency of *Bacillus* sp. CSK2 [16], and may enhance the biotechnological exploitation through heterologous overexpression and molecular optimization. The variation in sequence residues suggests that *Bacillus* sp. CSK2 keratinase is a novel protein. To date, a collection of bacterial species has been reported to exhibit keratinolytic potentials, but a few of these isolates have had their keratinase encoding genes successfully sequenced. Therefore, this augurs well for the biotechnology and industry, as extracellular secretion of important biomolecules by microbial producers represents a cost-effective process from the economic viewpoint.

## Conclusion

In this study, *Bacillus* sp. CSK2 keratinase production spiked at 48 h in a relatively cheap medium comprising chicken feathers as the only source of nitrogen. The produced keratinase showed optimal activity at pH 8.0 and 60 – 80 °C; with remarkable pH and thermal stability. The keratinolytic protease demonstrated remarkable stability (> 80%) after pretreatment with some selected commercial laundry detergents. This property suggests its potential application as bio-additive in detergents formulation. Furthermore, the putative keratinase encoding gene of *Bacillus* sp. CSK2 was successfully amplified and it showed high percentage similarity with keratinase genes from *B. cereus* group (*sensu* *lato*). Therefore, this substantively reaffirmed the keratinolytic property and extracellular keratinase secretion reported for *Bacillus* sp. CSK2. Evaluation of the amino acid sequence residues of the delineated gene showed that the keratinase is novel and structurally stable. This study serves a lead to unravel the biotechnological and industrial prospects of *Bacillus* sp. CSK2 keratinase; hence, scale-up production of this novel keratinase is imperative and would be focused on through molecular cloning and heterologous expression of the enzyme in a suitable industrial host.

## Methods

### Isolate and inoculum preparation

The keratinolytic bacterial isolate – *Bacillus* sp. CSK2 (MG215005) used for this research was previously isolated from dumpsite soil sample [16]. The pure bacterial culture maintained on powdered chicken feathers (PCF) constituted agar slant was used to inoculate basal salt medium (BSM) containing g/L of K_2_HPO_4_, 0.3; KH_2_PO_4_, 0.4; MgCl_2_, 0.2; CaCl_2_, 0.22; PCF, 10.0 and incubated for 48 h under agitation (130 rpm) at 30 °C. Subsequently, a loopful of the passaged culture was streaked on a freshly prepared PCF-agar plate containing BSM in addition of bacteriological agar (15.0 g/L). The plate was incubated for 18 h at 30 °C; thereafter, the cells were harvested into a microtube containing sterile saline (8.5 g/L NaCl). The optical density of the bacterial suspension was adjusted to 0.1 at 600 nm using spectrophotometer. The bacterial suspension served as the fresh inoculum for the fermentation process.

### Submerged fermentation and keratinase extraction

The production medium contained g/L of K_2_HPO_4_, 0.3; KH_2_PO_4_, 0.4; MgCl_2_, 0.2; CaCl_2_, 0.22, PCF, 7.5; sucrose, 2.0 in Erlenmeyer flasks (500 mL) containing 100 mL working volume. The flasks were sterilized in an autoclave (Already Enterprise Inc., Taiwan), and the initial pH was aseptically adjusted to 5.0 with the aid of JENWAY pH meter (Bibby Scientific Ltd., UK). The inoculation of the flasks was done with 2% (v/v) of the freshly prepared bacterial suspension and incubated for 48 h at 30 °C and 200 rpm. After the fermentation, supernatant was recovered using the centrifuge (Beckman Coulter, Inc. USA) at 15,000 rpm for 15 min and 4 °C. The supernatant served as crude keratinase and was utilized for the subsequent analytical studies without further purification.

### Keratinase activity assay

Keratinase activity was determined in the crude extract by using the previous [4], with slight modifications [15]. Briefly, 0.5 mL of 10 g/L of keratin azure (Sigma-Aldrich, USA) suspended in 0.1 M Tris-HCl buffer (pH 8.0) was mixed with 0.5 mL of enzyme solution. The mixture was incubated for 1 h at 60 °C and 220 rpm. The mixture was placed on ice-cold water for 10 min to stop the reaction, and then centrifuged at 15,000 rpm for 10 min to remove the insoluble substrates. The supernatant was evaluated for an azo dye release at 595 nm using SYNERGYMx 96 well micro plate reader (BioTek, USA). The control was buffer and enzyme solution treated under the same condition. One keratinase unit was defined as an amount of enzyme causing an increase in absorbance of 0.01 per hour under the standard assay protocol.

### Protein content quantitation

The concentration of protein in the cell-free crude extract was quantified with the Bradford method and the standard protein was bovine serum albumin [57].

### Thiol group determination

The thiol group content of the crude extract was quantitated spectrophotometrically (412 nm), following the measurement of yellow-colored 2-nitro-5-thiobenzoic acid (TNB) that formed upon reduction of 5,5-dithio-bis-2-nitro benzoic acid (DTNB) (Sigma-Aldrich, USA), by using the method of Ellman [58].

### Optimization of physico-chemical process conditions

The physico-chemical conditions were optimized using one variable at a time (OVAT) approach. The medium pH was carefully adjusted from pH 3.0 to pH 11.0 at interval of 1 unit in order to establish the optimal initial fermentation pH. Likewise, incubation temperature (25–40°C) and agitation speed (0–250 rpm) were varied at intervals of 5 °C and 50 rpm, respectively. The effect of supplementation of the fermentation medium with extra sources of carbon including sucrose, soluble starch, xylose, mannitol, glucose, fructose, maltose, galactose, lactose and sorbitol (Merck chemicals (Pty) Ltd., South Africa) was evaluated at final concentration of 1.0 g/L. The concentration of carbon source with the best stimulatory effect was varied from 0.8 to 10.0 (g/L). Additionally, the effect of different concentrations of chicken feather on keratinase production was investigated by varying the medium concentration from 1.0 to 17.5 (g/L). Finally, the time course study of keratinase activity of *Bacillus* sp. CSK2 was implemented at the optimized conditions for 120 h. Aliquots of the fermentation broth were periodically withdrawn (24 h interval) in an aseptic condition and used to evaluate some parameters including pH change, cell growth, keratinase activity, protein and thiol group concentrations.

### Keratinase characterization

#### Effect of pH on keratinase activity and stability

The optimum pH for keratinase activity was investigated using the following buffer solutions (0.1 M): sodium citrate (pH 5.0), potassium phosphate (pH 6.0–7.0), Tris-HCl (pH 8.0–9.0), and sodium bicarbonate-NaOH (pH 10.0–12.0) at 37 °C. Also, pH stability of the enzyme was assessed by pre-incubating the enzyme solution with different buffer solutions (pH 6.0–9.0) for 4 h at 37 °C; while aliquots were periodically withdrawn (1 h interval) and used to determine the residual activity under standard assay conditions.

#### Effect of temperature on keratinase activity and stability

The optimal temperature for keratinase activity was determined by carrying out enzyme assay at different incubation temperatures (30–80 °C). Thermal stability of the keratinase was assessed by subjecting the enzyme solution to thermal pretreatment at 50 °C, 60 °C and 70 °C for 120 min. Aliquots were periodically withdrawn (30 min interval), and the residual activity was determined under optimum assay conditions.

#### Effect of chemical agents on keratinase stability

The effect of some chemical agents (Sigma-Aldrich, USA) including ***protease inhibitors*** (5 mM): phenylmethylsulfonyl fluoride (PMSF), ethylene diamine tetraacetic acid (EDTA), 1,10-phenanthroline; ***reducing agents*** (5 mM): sodium thioglycolate (C_2_H_3_NaO_2_S), dithiothreitol (DTT), sodium sulfite (Na_2_SO_3_), 2-mercaptoethanol (2-ME); ***oxidizing agent*** (1%; v/v): hydrogen peroxide (H_2_O_2_); ***organic solvent*** (1%; v/v): dimethyl sulfoxide (DMSO), acetonitrile, propan-2-ol; ***non-ionic surfactants*** (1%; v/v): triton X-100, tween-80; and ***anionic surfactant*** (5 mM): sodium dodecyl sulfate (SDS) were pre-incubated with the enzyme solution for 1 h at 37 °C. After that, the residual enzyme activity was determined under the standard assay protocol. The enzyme solution pre-incubated with distilled water at similar conditions served as the control and was taken as 100%.

#### Effect of metal ions on keratinase stability

The effect of metal ions on keratinase stability was tested by pre-incubating the enzyme solution with various metal salts including LiCl, NaCl, AgCl, KCl, CaCl_2_, MgCl_2_, MnCl_2_, ZnCl_2_, CuCl_2_, FeCl_2_, HgCl_2_, BaCl_2_, CoCl_2_, AlCl_3_ and FeCl_3_ (Merck chemicals (Pty) Ltd., South Africa) at a final concentration of 5 mM for 1 h at 37 °C. Residual keratinase activity was determined under the optimum enzyme assay conditions. The enzyme solution pre-incubated with distilled water served as the control and was taken as 100%.

#### Effect of solid laundry detergents on keratinase stability

The impact of some selected commercially available laundry detergents on the crude keratinase was evaluated by following the method of Paul et al. [19]. Briefly, the solutions of solid laundry detergents which included Sunlight, Omo, Surf (Unilever, South Africa), Ariel (Procter and Gamble, South Africa) and Maq (Bliss Brands (Pty) Ltd., South Africa) were prepared with tap water to a final concentration of 7.0 mg/mL. The inherent enzymes of the various detergents were inactivated by heat treatment at 100 °C for 30 min. The enzyme solution was mixed with the preheated detergent solutions in a ratio of 4:1, and incubated for 60 min at 40 °C. Thereafter, the residual keratinase activity was measured under standard assay conditions. The enzyme solution that was pretreated with tap water only served as the control and was taken as 100%.

#### Keratinase gene amplification

The genomic DNA extraction from *Bacillus* sp. CSK2 was carried out following the previously described method [59]. The amplification of keratinase encoding gene was done by conventional polymerase chain reaction (PCR) using a set of oligonucleotides; *kerBNK1*F: TCATCTACTGATTACGTTCC and *kerBNK1*R: TTAAGAAGCTTTATTTTCTTG as forward and reverse primers, respectively. The pair of primers was designed based on the nucleotide sequence of keratinase-encoding gene of *B. thuringiensis* (KX155576) available in the GenBank. This isolate is phylogenetically related to *Bacillus* sp. CSK2. The PCR was carried out using 25 μL reaction mixture that consisted of 12.5 μL of OneTaq® Quick-Load® 2X master mix (New England Biolabs Inc., South Africa), 5.5 μL of nuclease-free water, 1 μL each of both forward and reverse primers and 5 μL of DNA template. The target gene amplification was done by using T100™ Thermal Cycler (Bio-Rad Laboratories Inc., Singapore), under the following cycling conditions: initial denaturation at 95 °C for 5 min, 35 cycles of denaturation at 95 °C for 30 s, annealing at 50 °C for 1 min, extention at 72 °C for 1 min, and then final extention at 72 °C for 5 min. The amplicons were electrophoresed on ethidium bromide stained 1.2% agarose gel (Merck chemicals (Pty) Ltd., South Africa), and subsequently, visualized under ultraviolet trans-illuminator (Uvitec, UK).

#### DNA sequencing and phylogenetic analysis

The PCR products were analyzed using the dideoxynucleotide chain termination method [60]. Briefly, the amplified products were subjected to a post PCR clean-up using the Nucleofast 96 well post PCR clean-up plate (Macherey Nagel GmbH & Co., Düren, Germany) on a Tecan EVO150 robotic workstation following the manufacturer’s protocol. Subsequently, the purified PCR products were sequenced with the BigDye Terminator V3.1 sequencing kit (Applied Biosystems) on an ABI3730XL DNA analyzer using a 50 cm capillary array and POP7 (Applied Biosystems) following the protocols supplied by the manufacturer. The PCR products were sequenced bi-directionally, to obtain a reliable sequence. The nucleotide sequences were analyzed using Geneious Prime V2020.1.1 (Biomatters Ltd., Auckland, New Zealand). BLAST search was conducted to retrieve similar sequences from the National Centre for Biotechnology Information (NCBI) (https://blast.ncbi.nlm.nih.gov/Blast.cgi). The multiple amino acid sequences alignment was conducted in BioEdit [61].

### Statistical analysis

Experiments were carried out in triplicates. The datasets obtained were submitted to analysis of variance and the degree of freedom was set at *P* < 0.05 significance level. The analysis was conducted in Statistical Package for Social Science version 23.

## Supplementary Information

**Additional file 1: Figure S1.** Gel picture of amplified keratinase gene from *Bacillus* sp. CSK2. Lane 1: DNA ladder (500 bp – 10,000 bp), lane 2: negative control, lanes 3–6: PCR amplified keratinase gene

## Data Availability

The results of the datasets analyzed during the current study were included in the manuscript and the nucleotide sequence of Bacillus sp. CSK2 and the amplified keratinase gene was submitted to the NCBI (https://blast.ncbi.nlm.nih.gov/Blast.cgi) through the accession numbers **MG215005** and **MT268136**, respectively. Any additional information is available from the corresponding author on reasonable request.

## Abbreviations

PCF: Powdered chicken feathers
BSM: Basal salt medium
DTNB: 5,5-Dithio-bis-2-nitro benzoic acid
TNB: 2-Nitro-5-thiobenzoic acid
PMSF: Phenylmethylsulfonyl fluoride
EDTA: Ethylene diamine tetraacetic acid
DMSO: Dimethyl sulfoxide
SDS: Sodium dodecyl sulfate
PCR: Polymerase chain reaction
pI: Isoelectric point
NCBI: National Centre for Biotechnology Information
