# Supplementary material for: Bacillus sp. CSK2 produced thermostable alkaline keratinase using agro-wastes: keratinolytic enzyme characterization
Source: BMC Biotechnol. 2020 Dec 14;20:65. doi: 10.1186/s12896-020-00659-2 (PMC7734832; doi:10.1186/s12896-020-00659-2)
Supplement: Supplementary file 1 — Additional file 1: Figure S1. Gel picture of amplified keratinase gene from Bacillus sp. CSK2. Lane 1: DNA ladder (500 bp – 10,000 bp), lane 2: negative control, lanes 3–6: PCR amplified keratinase gene [file 12896_2020_659_MOESM1_ESM.docx]

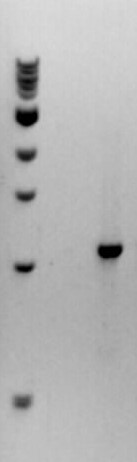


1500 bp

1104 bp

1000 bp

500 bp

1 2 3

**Fig S1** Gel electrophoresis of an amplified DNA region of *Bacillus* sp. CSK2 genome encoding the keratinase. The cropped gel picture showed lane 1: DNA ladder (500–10,000 bp), lane 2: negative control, lane 3: PCR product (1104 bp)
